# Supplementary material for: Mucosal Genes Encoding Clock, Inflammation and Their Mutual Regulators Are Disrupted in Pediatric Patients with Active Ulcerative Colitis
Source: Int J Mol Sci. 2024 Jan 25;25(3):1488. doi: 10.3390/ijms25031488 (PMC10855499; doi:10.3390/ijms25031488)
Supplement: Supplementary file 1 [file ijms-25-01488-s001.zip › ijms-2817332-supplementary.pdf]

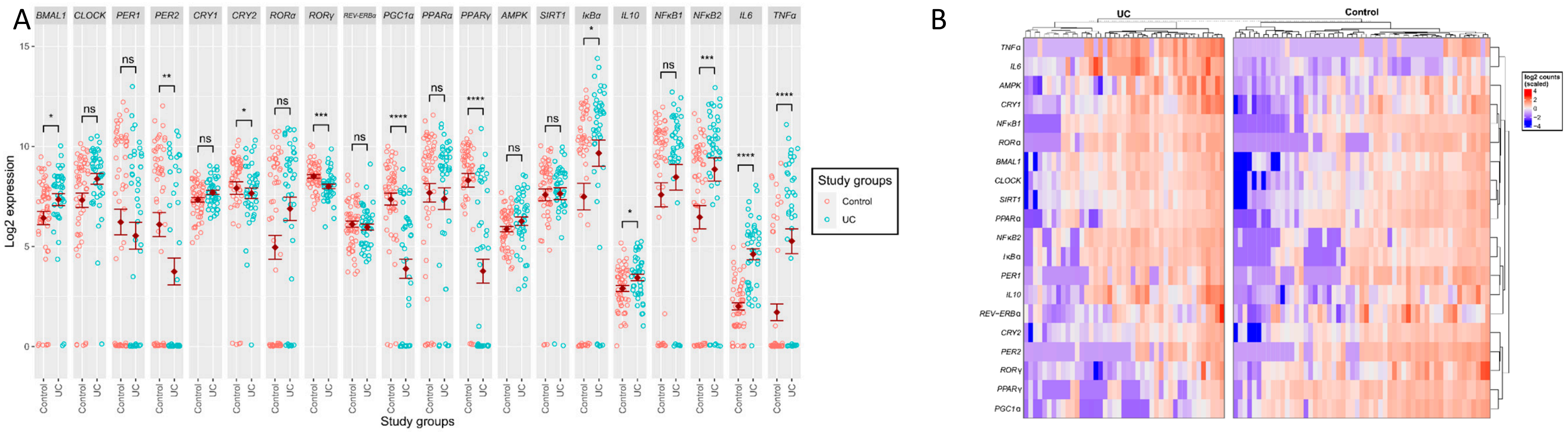

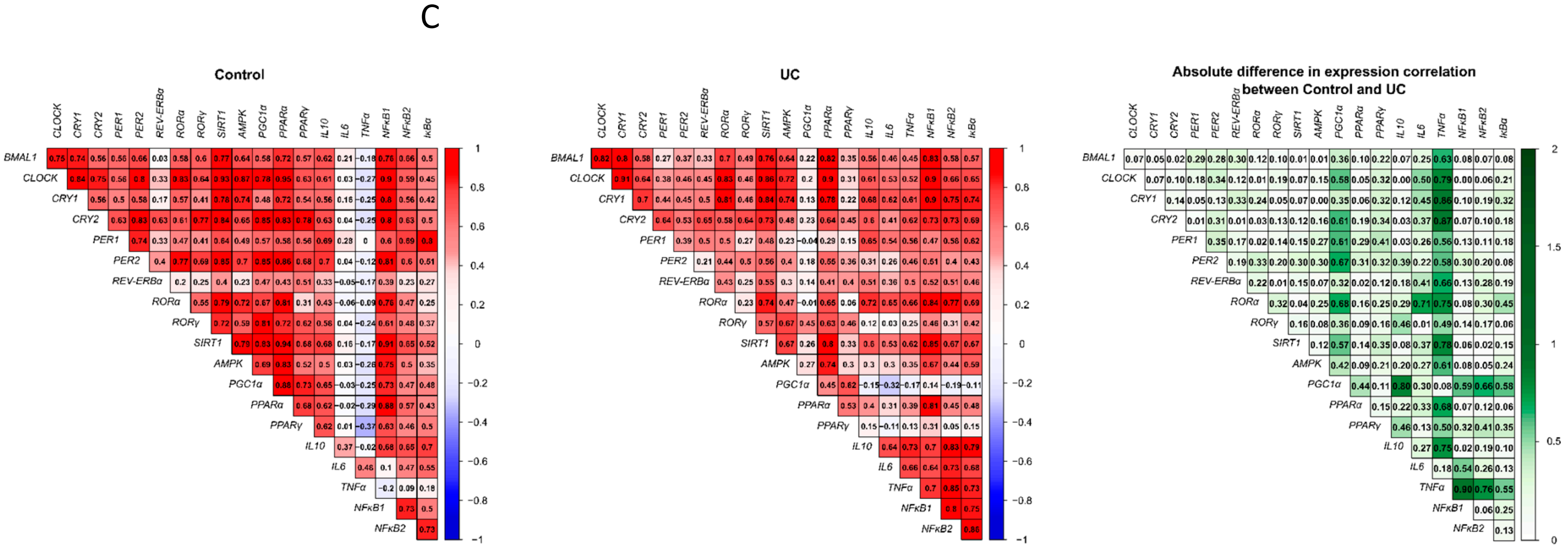

Figure S1: Expression and correlation of genes encoding clock, inflammation and their mutual regulators in rectal biopsies of active patients with UC. A. Expression level. B. Heatmap of gene expression. C. Correlation of gene expression between pairs of genes. The rightest panel is the absolute difference between the gene-pair correlation of controls and UC patients. Data are means ± SE. Asterisks denote significant differences: * is 0.05≥p≥0.01, ** is 0.01≥p≥0.001, * is 0.001≥p≥0.0001, **** p≤0.0001.**
